# Global Pseudo-Atrial Flutter on Electrocardiogram and the Importance of Clinical Correlation

**DOI:** 10.7759/cureus.35982

**Published:** 2023-03-10

**Authors:** Ashwin Jagadish, Shobha Hiremagalur

**Affiliations:** 1 Internal Medicine, East Tennessee State University James H. Quillen College of Medicine, Johnson City, USA; 2 Cardiology, Ballad Health CVA Heart Institute, Johnson City, USA

**Keywords:** parkinson’s disease, relevant physical examination, tremor, artifact, electrocardiogram (ecg/ekg)

## Abstract

Parkinson’s disease is a condition in which tremors, rigidity, bradykinesia, difficulties with sleep, autonomic symptoms, and mood disturbances can be present. We present an intriguing case in which such tremors appear as a global pseudo-atrial flutter on electrocardiogram (ECG). A 73-year-old Caucasian female presented to the cardiology clinic for management of atrial flutter diagnosed by ECG in a primary care setting. In the cardiology clinic, the physical examination of the patient revealed bilateral upper extremity resting tremors. The ECG machine initially read the findings as “atrial flutter.” However, immobilization of the patient’s arms bilaterally resulted in a normal sinus rhythm. Repeated ECGs when the arms were relaxed and when the arms were immobilized resulted in findings consistent with pseudo-atrial flutter and normal sinus rhythm, respectively. Considering tremors as a source of artifact on electrocardiogram in patients with tremors and using corrective measures are critically important to prevent misdiagnosis, unnecessary testing, and potentially harmful treatments. This case underscores the importance of educating healthcare team members about tremor-induced artifacts in patients with tremors to avoid misdiagnosis based on ECG readings.

## Introduction

Parkinson’s disease is a neurodegenerative condition impacting a minimum of 1% of individuals above the age of 60 [1]. The condition is characterized by a reduction in the level of dopaminergic neurons within the substantia nigra [1]. The first sign of Parkinson’s disease is often tremors and is later accompanied by rigidity and bradykinesia [1]. Other associated signs include impaired olfaction, difficulty with sleep, autonomic symptoms, mood disturbances, and constipation [1].

Atrial flutter is a cardiac rhythm disturbance in which the atrial rate is noted to be around 300 beats/minute, while the ventricular rate is around 150 beats/minute [2]. Patients can be asymptomatic or experience dyspnea, palpitations, fatigue, and lightheadedness [2]. The risk of stroke is comparable to that of atrial fibrillation, so the identification of causes and treatments is vital [2].

Artifacts, electrical alterations unrelated to cardiac electrical activity, can be seen on electrocardiogram (ECG) and result in the distortion of the waves [3]. Artifacts can be due to motion: Parkinson’s disease, anxiety, medication-induced, shaking, or sudden movements [3].

In this case, we present an interesting case of Parkinsonian tremor presenting as a global pseudo-atrial flutter on electrocardiogram. It is important to consider not only the ECG but also the patient’s physical examination to avoid unnecessary treatments for the patient.

## Case presentation

**Figure 1:**
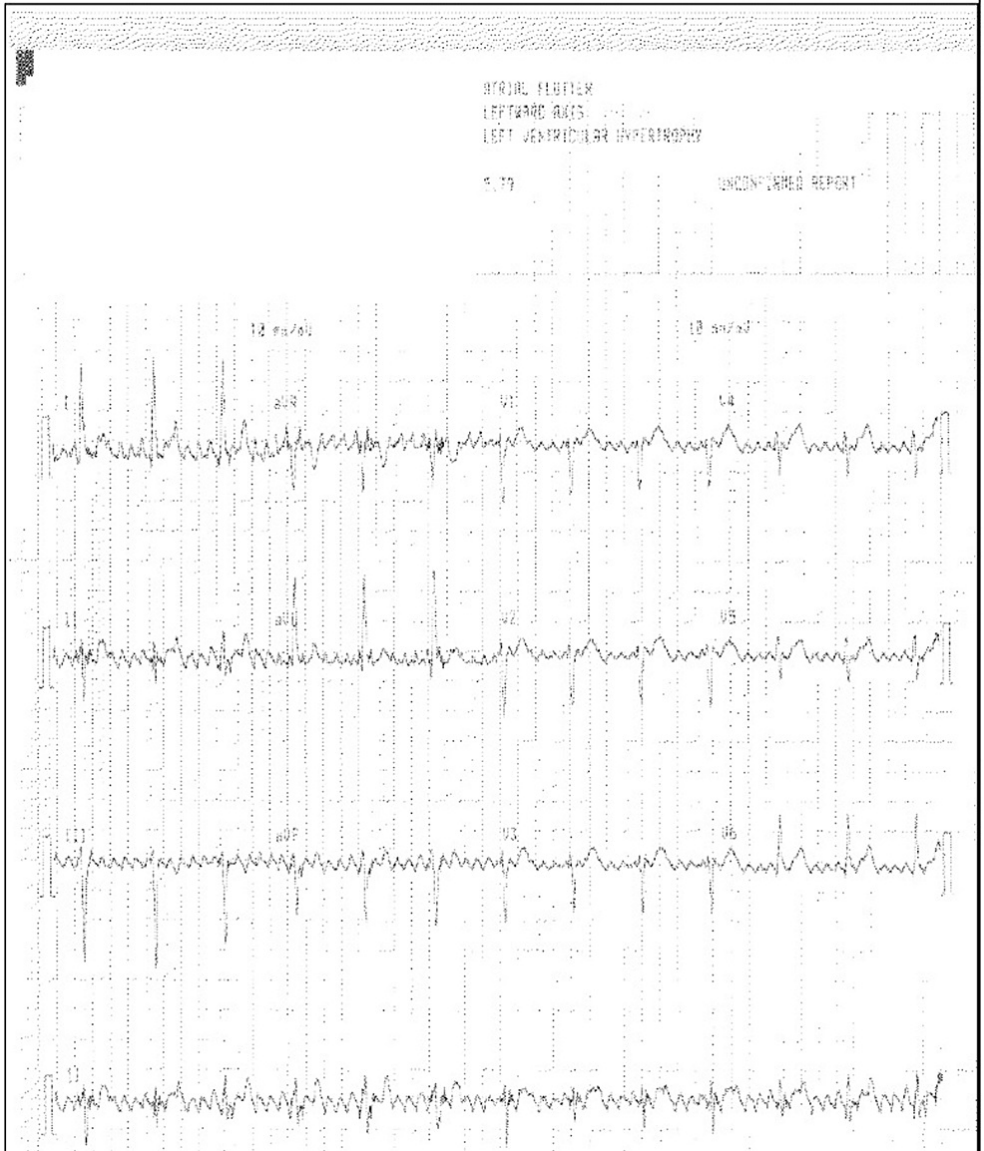
Electrocardiogram obtained from the primary care physician’s office with machine reading “atrial flutter”

A 73-year-old Caucasian female with depression, anxiety, type II diabetes mellitus, hypertension, and Parkinson’s disease presented to the cardiology clinic to establish herself as a new patient. Three weeks prior, she presented to her primary care provider to follow up on her depression and anxiety. During this visit, a routine 12-lead electrocardiogram (ECG) was obtained, and the machine reading indicated “atrial flutter” (Figure 1). The ECG was repeated again during that visit, and the machine reading also indicated “atrial flutter.” The primary care provider initiated her on apixaban 5 mg to be taken twice daily for stroke prevention and referred her to the cardiology clinic for further management.

**Figure 2:**
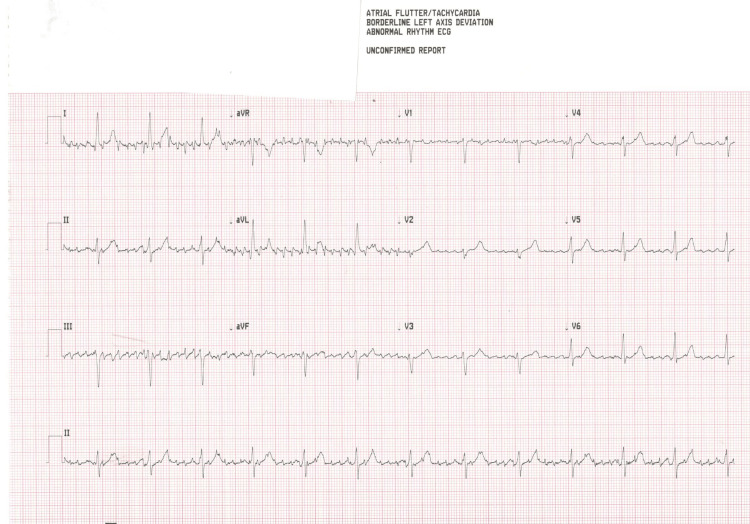
Electrocardiogram obtained in the cardiology clinic with machine reporting “atrial flutter”

**Figure 3:**
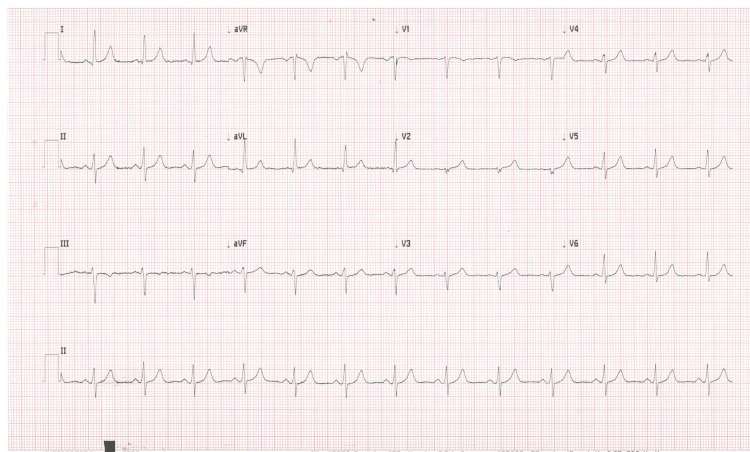
Electrocardiogram obtained in the cardiology clinic with the patient’s arms completely immobilized; sinus rhythm is noted

**Figure 4:**
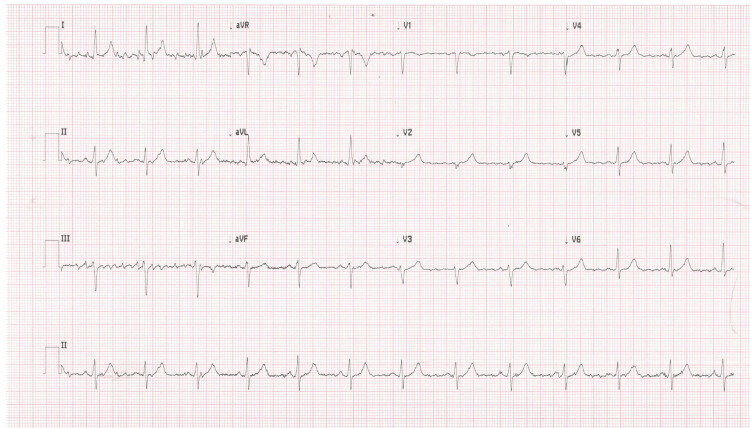
Electrocardiogram obtained in the cardiology clinic with the patient’s arms partially immobilized; pseudo-atrial flutter was noted in some, but not all, leads

At the cardiology clinic, the patient’s blood pressure was 144/78 mmHg, heart rate was 86 beats per minute, and oxygen saturation was 98% on room air. She endorsed anxiety but denied episodes of palpitations. Her cardiac physical examination indicated a regular rate and rhythm. She was noted to have bilateral upper extremity resting tremors, with the tremors more prominent on the left extremity than the right extremity. The intensity of her tremors fluctuated throughout the course of her interview depending on the position of her arm. An ECG was conducted on the patient, indicating the presence of “atrial flutter” (Figure 2). Tremors were suspected to be impacting the ECG results; as a result, the patient was asked to press her hands firmly down on the examination table to minimize movement. An ECG taken in this style resulted in a normal sinus rhythm (Figure 3). The ECG was then repeated with her arms completely relaxed, and findings of global pseudo-atrial flutter were noted once again. Subsequently, her arms were partially immobilized on the examination table, resulting in the pseudo-atrial flutter in some, but not all, leads (Figure 4). However, when her arms were fully immobilized, the repeat ECG indicated normal sinus rhythm without any “flutter” waves in any leads. Thus, it was determined that the ECG findings were actually pseudo-atrial flutter induced by Parkinsonian tremors and not true atrial flutter. Members of the healthcare team were then educated on the importance of physical examination findings and immobilization of extremities while conducting ECGs on patients with tremors.

## Discussion

Our patient was initially diagnosed with atrial flutter at her primary care provider’s office, initiated on apixaban therapy, and referred to the cardiology clinic. At the cardiology clinic, ECG findings of sharp “P” waves with varying “P” wave morphology in the same lead prompted questioning of the diagnosis of atrial flutter. Additionally, physical examination findings of bilateral upper extremity resting tremor prompted further evaluation. When the patient’s arms were immobilized, the ECG resulted in normal sinus rhythm, indicating that the machine-reported atrial flutter was merely global pseudo-atrial flutter, an artifact.

Common sources of artifacts include other electronic devices present within the examination room, tremors, and misplacement of electrodes [3]. These artifacts can present as atrial fibrillation, atrial flutter, or ventricular tachycardia on ECG [3].

Erroneous diagnosis can result in hospitalization, initiation of anticoagulation, and electrophysiological procedures [4,5]. Given the serious consequences of misdiagnosis due to artifacts, we recommend careful examination of the patient. Consideration of physical examination findings, including tremors, can help differentiate between genuine medical conditions and artifacts. This differentiation can prevent patients from undergoing potentially unnecessary and harmful treatments. Educating members of the healthcare team about ECG findings in patients with tremors may be beneficial to patient safety, as an inaccurate diagnosis can lead to unnecessary treatments.

## Conclusions

Patients with Parkinson’s disease often present with tremors, which can result in the distortion of ECG results. Consideration of physical examination findings is important to determine whether ECG findings are genuine or artifacts. Failure to make this determination can result in potentially unnecessary and harmful treatments for the patient. Education of healthcare team members, especially those performing the ECG, is crucial to prevent potential misdiagnosis based on ECG interpretation.
